# Draft Genome Sequence of Geobacillus stearothermophilus Strain K4E3_SPR_NPP, Isolated from Kasol Hot Spring, Himachal Pradesh, India

**DOI:** 10.1128/mra.00194-22

**Published:** 2022-05-19

**Authors:** Seema Rodge, Niranjan Patil

**Affiliations:** a Department of Microbiology, MES Abasaheb Garware College, Pune, Maharashtra, India; Montana State University

## Abstract

Geobacillus stearothermophilus strain K4E3_SPR_NPP is a thermophilic bacterium growing optimally at 70°C. Here, we present the draft genome sequence of Geobacillus stearothermophilus strain K4E3_SPR_NPP, which was isolated from Kasol Hot Spring (72.3°C), Himachal Pradesh, India (32°0′46.35″N, 77°19′0.4908″E).

## ANNOUNCEMENT

The organism formerly known as Bacillus stearothermophilus was transferred to a new genus, *Geobacillus*, by T. Nazina et al. in the year 2001 (1). Kasol Hot Spring is located in Himachal Pradesh, India (32°0′46.35″N, 77°19′0.4908″E). Water was collected from the hot spring in a sterile screw cap bottle and preserved at 4°C for further processing (2). Thermophilic bacteria were isolated and incubated at 70°C for 24 h using nutrient agar medium.

For genomic DNA extraction, a single colony was selected. DNA was extracted using the DNeasy PowerSoil Pro kit from Qiagen (catalog number 47017). The genomic DNA (gDNA) was sequenced using the Illumina NovaSeq 6000 instrument. A gDNA library was prepared as per the TruSeq DNA Sample Preparation Guide. The fastq reads were trimmed using Trim Galore (http://www.bioinformatics.babraham.ac.uk/projects/trim_galore/) (3). The trimmed reads were assembled using Unicycler v0.5.0 (https://github.com/rrwick/Unicycler) (4). The primary assembly was subjected to secondary assembly using CONTIGuator (https://combo.dbe.unifi.it/contiguator), with the closest reference genome as the reference background (5). The closest reference, based on a BLAST search of the 16S rRNA sequence similarity, was identified as Geobacillus stearothermophilus ATCC 12980 (GenBank accession number JYNW00000000.1). The secondary assembly was subjected to PlasmidFinder v2.1 to find the plasmids, if any (https://cge.cbs.dtu.dk/services/PlasmidFinder/) (6, 7). The genome was annotated using the NCBI Prokaryotic Genome Annotation Pipeline (PGAP) v5.3 (8). The CRISPRCasFinder Web server was used to detect CRISPR arrays (9). The taxonomic assignment was obtained using the Genome Taxonomy Database (GTDB) toolkit (GTDB-Tk) v1.7.0, release R06-RS202 (https://gtdb.ecogenomic.org/) (10–16). The Genome-to-Genome Distance Calculator (GGDC) v3.0 (https://ggdc.dsmz.de/ggdc.php#) was used to calculate the digital DNA-DNA hybridization (dDDH) values (17). Default parameters were used for all software unless otherwise noted.

The genome of the thermophilic isolate is 2,993,965 bp long. In all, 19.8 million paired-end reads having a length of 150 bp were generated, with 1,024× genome coverage. The genome was assembled into 95 contigs with an *N*_50_ value of 2,865,931 bp and 52.67% GC content. One plasmid was found in the secondary assembly. NCBI PGAP was used to identify 3,223 genes, with 2,955 protein-coding sequences and 3,117 coding DNA sequences (CDSs). Furthermore, it was used to identify 86 tRNA sequences, 7 5S rRNA sequences, 1 complete 16S rRNA sequence, 6 23S rRNA sequences, and 5 noncoding RNAs (ncRNAs). A type IIC CRISPR-Cas system was identified, and the genome showed the presence of 5 CRISPR arrays and 43 spacers. The analysis using GTDB-Tk v1.7.0 identified the isolate as Geobacillus stearothermophilus, with the following classification: domain *Bacteria*, phylum *Firmicutes*, class *Bacilli*, order *Bacillales*, family *Anoxybacillaceae*. Using GGDC v3.0, the generalized linear model (GLM)-based DDH estimate was calculated as 74.0% and the distance as 0.0308 with Geobacillus stearothermophilus ATCC 12980 (GenBank accession number JYNW00000000.1). As the DDH is not greater than 79%, there is a possibility that the isolate belongs to a new subspecies (18).

### Data availability.

The whole-genome sequencing project for Geobacillus stearothermophilus strain K4E3_SPR_NPP has been deposited at DDBJ/ENA/GenBank under the accession number JAKLOQ000000000. The version described in this paper is version JAKLOQ020000000. The raw sequencing reads have been deposited at GenBank under the SRA accession number SRX14002899. The BioSample accession number is SAMN25211115, and the BioProject accession number is PRJNA799873.
